# Implementation of the CHIldren with acute COugh (CHICO) intervention to improve antibiotics management: a qualitative study in primary care

**DOI:** 10.3399/BJGP.2023.0330

**Published:** 2024-04-30

**Authors:** Clare Clement, Jenny Ingram, Christie Cabral, Peter S Blair, Alastair D Hay, Penny Seume, Jeremy Horwood

**Affiliations:** Centre for Appearance Research, University of the West of England (UWE Bristol), Bristol.; Centre for Academic Child Health, Bristol Medical School, Population Health Sciences, University of Bristol, Bristol.; Centre for Academic Primary Care, Bristol Medical School, Population Health Sciences, University of Bristol, Bristol.; Centre for Academic Child Health, Bristol Medical School, Population Health Sciences, University of Bristol, Bristol.; Centre for Academic Primary Care, Bristol Medical School, Population Health Sciences, University of Bristol, Bristol.; Centre for Academic Primary Care, Bristol Medical School, Population Health Sciences, University of Bristol, Bristol.; Centre for Academic Primary Care, Bristol Medical School, Population Health Sciences, University of Bristol, Bristol.

**Keywords:** algorithms, antibiotics, child, cough, hospitalisation, primary care

## Abstract

**Background:**

Childhood respiratory tract infections (RTIs) are common and can lead to unnecessary antibiotic use and antimicrobial resistance. The CHIldren with COugh (CHICO) intervention incorporates a clinician-focused algorithm (STARWAVe) to predict future hospitalisation risk, elicitation of carer concerns, and a carer-focused personalised leaflet recording treatment decisions and safety-netting information.

**Aim:**

To examine the implementation of the CHICO intervention by primary care clinicians.

**Design and setting:**

A qualitative study with primary care clinicians in England taking part in the CHICO randomised controlled trial.

**Method:**

Interviews explored the CHICO intervention’s acceptability and use. Clinicians from a range of practices with high and low antibiotic dispensing rates were recruited. Normalisation process theory underpinned data collection and thematic analysis.

**Results:**

Most clinicians liked the intervention because it was quick and easy to use, it helped elicit carer concerns, and reassured clinicians and carers of the appropriateness of treatment decisions. However, clinicians used it as a supportive aid for treatment decisions rather than as a tool for behaviour change. The accompanying advice leaflet helped explain treatment decisions and support self-care. The intervention did not always align with clinicians’ usual processes, which could affect use. Increased familiarisation with the algorithm led to reduced intervention use, which was further reduced during the COVID-19 pandemic as a result of changes to practice and remote consultations.

**Conclusion:**

Clinicians found the CHICO intervention useful to support decision making around antibiotic prescribing and it helped discussions with carers about concerns and treatment decisions. The intervention may need to be adapted to align more with clinicians’ consultation flow and remote consultations.

## Introduction

Antimicrobial resistance is recognised as one of the most pressing global public health threats of our time.^1^ Around 80% of all antibiotics are prescribed in primary care,^2^ with approximately 50% of prescriptions in this setting being unnecessary.^3^ Respiratory tract infections (RTIs) in children present a major primary care challenge because they are common and costly, and ongoing uncertainty regarding diagnosis and management is a major driver of antibiotic prescribing.^4^^,^^5^ Improved identification of children at low risk of future hospitalisation could increase the confidence not to prescribe antibiotics. To address this, the CHIldren with COugh (CHICO) cluster randomised controlled trial (RCT) (trial registration number: ISRCTN11405239)^6^^,^^7^ evaluated whether unnecessary antibiotic prescribing could be reduced by providing an intervention to support clinicians’ decision making for antibiotic prescribing in children with RTIs in primary care.^8^^,^^9^

In the RCT, the CHICO intervention did not reduce overall antibiotic dispensing for children presenting with RTIs. Neither did it increase hospitalisation rates for RTIs in the intervention practices compared with usual practice. Sensitivity and subgroup analyses indicated decreased dispensing rates in the intervention arm for older children, for practices restricted to one site, for practices with proportionally fewer nurse practitioners, and for practices in less deprived areas. A post hoc analysis also indicated reduced antibiotic dispensing levels in the intervention arm in the pre-pandemic period. Among 121 practices with at least 1 month of intervention data, there were 11 944 observed uses. The median number of times the intervention was used for the 115 practices with 12 months of data was 70 uses at each practice, with an interquartile range of 9 to 142. Full results are reported elsewhere.^6^

Whether an intervention is valued and what clinicians need to put it into practice can influence whether and how an intervention is used in primary care.^10^^,^^11^ This article describes the CHICO RCT nested qualitative study, which aimed to explore the use of the intervention and how it was embedded within usual practice.

**Table:** How this fits in

The CHIldren with COugh (CHICO) intervention was designed as an aid to reduce unnecessary antibiotic prescribing for respiratory tract infections in children in primary care. However, the intervention did not significantly reduce overall antibiotic dispensing. GPs and nurses initially welcomed the intervention but faced difficulties integrating it into their usual consultation flow, leading to reduced use over time. The CHICO intervention may still be valuable in supporting decision making and discussions, but it needs adjustments for better integration into clinicians’ workflow and remote consultations.

## Method

### Study design

The CHICO trial was a two-arm (intervention versus usual care) efficient, pragmatic, open-label RCT in 294 primary care practices (144 intervention and 150 control) in England. The intervention was designed to reduce antibiotic dispensing without impacting hospital attendance in children aged 0–9 years with acute cough and RTIs over 12 months.^8^ The CHICO trial was conducted between October 2018 and September 2021, and included the COVID-19 pandemic, which began in March 2020 and was still ongoing in September 2021.

The intervention had three components: eliciting explicit carer concerns during the consultation; a clinician-focused algorithm (STARWAVe)^12^ to identify children at very low risk of future hospitalisation in whom antibiotics could be safely withheld; and a carer-focused personalised leaflet co-designed with carers recording decisions made at the consultation, addressing common concerns, and providing safety-netting information (see Supplementary Figure S1 for details).^9^

The intervention was triggered when a child of 0–9 years presented and the healthcare professional received a ‘soft’ (that is, a reminder) pop-up screen alert asking if the child was presenting with an RTI and providing the option to open the CHICO intervention. Clinicians could also initiate the intervention using specific EMIS (an electronic patient record management system) RTI codes.

Clinicians involved in implementing the CHICO intervention were invited to take part in semi-structured interviews to explore the use of the intervention, how it was embedded into practice, and whether it was acceptable.

Interviews were conducted in two phases (during the pilot period and after 12 months’ intervention period), with findings from the pilot phase used to make changes in the main trial.

Data collection and analysis were informed by the normalisation process theory (NPT), which proposes that intervention implementation is dependent on four criteria (Box 1).^13^ These four constructs were used to guide data collection and develop themes during analysis.

**Box 1.** Normalisation process theory constructs and how they are applied to the CHICO intervention

**Core construct**	**Explanation**	**How applied to CHICO**
Coherence	The meaning of the intervention to people and sense-making work they do to operationalise new practices. Individuals’ clarity regarding the purpose of the intervention	How do clinicians understand the CHICO intervention and its purpose?
Cognitive participation	Whether there is buy-in from the people who are responsible for implementing the intervention. Work that individuals and organisations necessarily do to enrol individuals to engage with the intervention	Do clinicians engage with the CHICO intervention and how is this achieved?
Collective action	The work that people do to put new interventions and their components into operation. The work that individuals must do to make the intervention function	What do clinicians do to use CHICO in their practice?
Reflexive monitoring	Participants’ reflection or appraisal of the intervention	How do clinicians reflect on their use of the CHICO intervention? How do they think it can be improved for future use in this context?

### Sampling and recruitment

Purposive, maximum variation sampling was used to capture variation in views and experience of clinicians (GPs and practice nurses) from a range of practices. Clinicians from 56 of the 144 intervention practices in the RCT were invited to take part in an interview via email. Practices were selected based on multiple characteristics — those with large and small patient list size, with high and low antibiotic dispensing rates, and serving areas of high and low sociodemographic deprivation, with data taken from clinical commissioning groups (CCGs) — to facilitate a comprehensive understanding of any complexity and variability across practices. The socioeconomic status of practices was estimated using the English indices of deprivation 2019.^14^ The sample size was determined by data saturation (additional data provided limited additional insights)^15^ and assessed as analysis progressed. All clinicians who expressed an interest to be interviewed took part.

### Data collection

Telephone interviews were conducted by an experienced social science researcher and lasted between 15 and 37 minutes, with an average time of 25 minutes. Interviews were conducted between March and September 2019 during the pilot trial phase, and between November 2019 and February 2021 (the main trial phase after practices had been using the intervention for 12 months). A flexible topic guide was used to guide interview questioning but allowed participants to present unanticipated issues (see Supplementary Box S1 for details). Similar topics were covered in both phases, with the impact of the COVID-19 pandemic added after March 2020. Audio-recorded verbal consent was gained before the interviews.

### Data analysis

Interviews were audio-recorded, transcribed, anonymised, and imported into NVivo (version 10/11). The pilot data underwent rapid analysis,^16^ influencing the implementation of the intervention (see Supplementary Box S2 for details). Thematic analysis^17^ was subsequently applied to all data, using iterative and deductive coding guided by NPT constructs. Initial codes were iteratively developed and then deductively organised based on NPT constructs. Three transcripts (one from the pilot phase and two from the main phase) were independently coded by experienced researchers for initial codes and groupings. These codes were then applied to remaining transcripts, with ongoing refinement. Collaboratively, three researchers developed themes within NPT constructs. Preliminary findings were discussed with the multidisciplinary trial management team for trustworthiness and enhanced understanding.

## Results

Overall, 26 clinicians (20 GPs and six practice nurses) were interviewed from 24 practices (range of one to two clinicians per practice) and 13 CCGs (Table 1). Ten clinicians (eight GPs and two practice nurses) were interviewed from eight practices during the pilot phase. Findings are presented for each of the NPT constructs, illustrated with anonymised quotations (pilot interview quotations are indicated).

**Table 1.** Participant characteristics

**Characteristic**	**Participants, *n***	

	**Nurses (*n* = 6)**	**GPs (*n* = 20)**
**Years’ experience**		
0–5	1	1
6–10	2	5
11–15	0	6
16–20	1	0
21–25	1	7
≥26	1	1

Above CCG median practice patient list size	4	10
Below CCG median practice patient list size	2	10

Above CCG median antibiotic dispensing rate	3	8
Below CCG median antibiotic dispensing rate	3	12

**Practice deprivation score**		
High	1	6
Medium	2	7
Low	3	7

*CCG = clinical commissioning group.*

### Coherence (understanding the purpose of CHICO)

Clinicians welcomed the CHICO intervention as it aimed to help with perceived carer concerns about not receiving antibiotics, it aligned with existing strategies and efforts to reduce unnecessary antibiotic prescribing, and clinicians believed it would fit within their usual practice:

*‘It was something that we were interested in doing … We do see lots of children with coughs and colds, and some parents are generally concerned … some do also expect antibiotics if it’s had a chest cough for a certain period*.*’*

(GP 14)

### Cognitive participation (engagement with CHICO)

Clinicians felt they were well prepared for using the intervention and found training guides and the ability to practise using a test patient useful:

*‘I think* [we were] *really well prepared. The training results are really good but having a test patient was really good*.*’*

(GP 11)

### Collective action (using CHICO in practice)

Most clinicians liked the intervention and used it as a supportive aid in consultations. It was a way of reassuring themselves and carers of the appropriateness of treatment decisions:

*‘It’s very reassuring for the professional and, of course, when you’ve printed out the leaflet that is the scoring we have done; it is very reassuring for parents as well*.*’*

(GP 16)

#### Launching CHICO

During the pilot phase, clinicians were reminded to use the intervention via an electronic patient record system ‘pop-up’, which was triggered for all children under the age of 10 at the start of the consultation. Following feedback, this was modified for the main trial, with clinicians having the choice of the early pop-up or of launching it later in the consultation when typing in ‘cough’. In the main trial, some practices retained the pop-up to help remind clinicians to use the intervention:

*‘It seems the hard pop-up was useful, but it comes up too early. So, it comes up before you know what’s actually wrong with the child*.*’*

(Nurse 8, pilot)

*‘We’ve kept the automatic launch on, even though it is a bit annoying … We thought the best way to try and get people to use it and remember it for the whole year was to keep it as automated.’*

(GP 13)

#### Prognostic algorithm

Most clinicians liked the signs and symptoms template, which they found easy to use without adding any more time to consultations:

*‘I think that’s quite straightforward … I thought it was good, it was easy to use.’*

(Nurse 6, pilot)

However, some clinicians felt the template did not capture all the required information, meaning that they needed to make additional entries in the patient’s record or *‘moving between two screens’* (GP 11), which could be problematic:

*‘I found I either use the template and then probably it was a bit sketchy history, or I had to then go into the patient’s note or save the template, and then do a history or presenting complaints, and I found that made it a bit more disjointed*.*’*

(GP 19)

Clinicians liked that the template helped to elicit carer concerns, which were important but could easily be forgotten:

*‘I suppose I wouldn’t necessarily ask “what are you particularly worried about?” directly.’*

(Nurse 17)

The usefulness of the prognostic algorithm depended on the severity of the child’s symptoms. It was most useful with children who were ‘borderline’ cases for hospitalisation or prescribing antibiotics:

*‘I personally would* [have] *used it* [prognostic algorithm] *more for the borderline ones.’*

(GP 14)

#### Letter/advice leaflet

The carer advice leaflet was reported to be the most useful intervention component and clinicians liked it as a *‘good safety netting tool’* (GP 19), a way of facilitating conversations with carers, and reinforcing the clinician’s decision not to prescribe antibiotics:

*‘That* [advice leaflet] *was quite helpful to feel you give the parents a little bit more understanding and information of what they’re looking out for before perhaps they worry or to help reduce their anxiety over their children’s coughs.’*

(Nurse 6, pilot)

*‘So, if there was a feeling that it was going to be a difficult consultation to try and steer them* [parents] *away from antibiotics based on the clinical assessment, then that would be a really good adjunct tool for that*.*’*

(GP 25)

GPs and nurses felt that carers were more satisfied with being given a leaflet that explained the clinician’s decision and having information they could take away with them:

*‘I think that it makes patients feel more satisfied that they’re not going away empty handed. They’ve been given something, and I feel kind of what I’ve said to them had been enhanced by going away with a leaflet*.*’*

(GP 11)

Nurses found being able to give the carers the leaflet particularly useful, as they felt they faced increased scrutiny and pushback from carers if they did not prescribe antibiotics:

*‘What we find quite often as an ANP* [advanced nurse practitioner] *is, if we refuse them antibiotics, then they go and make an appointment with the doctor and get antibiotics. So, you know, you’re always aware in the back of your mind that that kind of thing is going to rumble on … they’ll just go and keep seeing people until they get what they want … I would say that it gave us that extra back-up to say no*.*’*

(Nurse 24)

As with the prognostic algorithm, the advice leaflet was seen to be more useful for children considered to be ‘borderline’ for hospitalisation or prescribing antibiotics:

*‘Especially when there is a borderline whether to go to the hospital or not and the score is a bit on the lesser side and parents are not keen to go to the hospital and at that time, this has particularly helped. The leaflet you’re giving them the clear-cut advice of when to go and when to seek advice*.*’*

(GP 23)

#### Challenges with CHICO in practice

Several challenges were highlighted that led to reduced use of the intervention or selective use of some of its components. Clinicians reported difficulties aligning the intervention with their usual consultation practice. The use of the algorithm to support decision making and providing carers with the letter and advice leaflet required clinicians to engage with the computer and patient record throughout the consultation. However, some would usually complete the record at the end of the consultation or after the patient had left as they liked to focus on the patient during the consultation. This led some clinicians to stop using the intervention; however, in some cases, clinicians did provide carers with pre-printed non-personalised advice leaflets:

*‘I do my typing up at the end of the consultation so it* [intervention] *doesn’t alter my thought processes, “am I going to prescribe them antibiotics or not?” I have already made that decision from taking the history and doing the examination … It doesn’t actually give you the scoring until you click ‘save’ so that pop-up comes right up right at the end.’*

(GP 3, pilot)

*‘Most of us find that it gets in the way of our consultation and so, therefore, we don’t use it, but we like the leaflet, and we give that out.’*

(GP 11)

*‘Unfortunately, the leaflet thing probably got a little bit overlooked because you do the whole template, finish the consultation with the patient and then they would go and then you’d finish writing up your notes … and then up comes the “would you like to print a leaflet?” and it’s “oh, I’ve forgotten to do that”.’*

(Nurse 17)

Some practices conducted their consultations remotely, which meant assessing the clinical symptoms required for the prognostic algorithm was challenging. This was more of an issue with telephone consultations because some symptoms could still be assessed using video where the child could still be seen and heard:

*‘Can assess using video, can see the child … breathlessness, wheezing.’*

(GP 18)

*‘We do a lot of video consultations as well, then you can see whether the child’s running around and what they’re doing, so yes it could easily be adapted I would have thought.’*

(Nurse 20)

It was also difficult to provide carers with personalised printed leaflets in some practices, either because of the remote consultation or because of printing issues. However, some clinicians had found ways around this, including using non-personalised pre-printed versions of the leaflet provided by the study team, saving a pdf version that could then be printed off without using the intervention, and emailing or texting the leaflets to carers:

*‘Often, I would just give them a nice … they were very attractive leaflets and a bit more striking than the black and white paper printout.’*

(GP 19)

*‘We’ve started to email the leaflet to patients … using a text messaging service … So, we have used the leaflets via telephone consultation as well, so you can do that so that bit is good.’*

(GP 22)

#### Frequency of use

How often clinicians used the CHICO intervention was variable, with some reporting frequent use early on, but reduced use over time. This could depend on clinicians remembering to use it, how busy the practice was, and increased familiarity with the algorithm outcomes:

*‘In the latter months we sometimes would forget to do the CHICO template … The more you use it, the more you get used to it, and you get a feel of what the score might be and what the outcome might be.’*

(GP 14)

#### Perceived impact on prescribing behaviour

Some clinicians believed the intervention influenced their prescribing behaviour. However, others believed that it supported rather than changed their prescribing decisions, and did not change their behaviour:

*‘I’m not sure it massively did* [affect prescribing]*. Perhaps not directly I would say … We probably went on the history and the physical examination.’*

(Nurse 17)

*‘The main thing we used it for was safety netting and we do that anyway, so it’s really just enhancing. Not like we’re saying “okay, we are going to ignore everything in front of us because CHICO is telling us to do this”. It really just fits in with what we do anyway.’*

(GP 11)

#### Use during the COVID-19 pandemic

Changes in practice pathways, such as increased use of remote consultations and nurse triaging, and the use of COVID-19 protocols, led to reduced use of the CHICO intervention during the pandemic. Anyone presenting with a cough was assessed for potential COVID-19 infection and referred for a COVID-19 test:

*‘COVID took over, I think that completely took over … and also cough just took on a whole new meaning.’*

(Nurse 17)

The need to conduct consultations in restricted spaces with no computers meant that clinicians were unable to use the CHICO prognostic algorithm and they could not print out the personalised letters or use pre-printed leaflets:

*‘The problem was that we were seeing patients with coughs and temperatures in a red room. So, we’d cleared everything from that clinical room … so we didn’t have the leaflets readily available and also we weren’t logging onto that computer … so, I don’t think we’d probably used it quite as much during COVID.’*

(GP 19)

*‘Part of the reason that we wouldn’t use it during COVID is that we’re seeing our patients … outside in the car park, so we don’t have our computer in front of us.’*

(GP 15)

The increased use of remote consultations during the COVID-19 pandemic further highlighted the challenges discussed. However, having used the intervention during remote consultations during this period, clinicians did perceive some benefits to using it remotely, including less need to focus on a face-to-face consultation:

*‘It fits more naturally with remote working because it’s easier to get whatever you need on the* [computer] *screen, and you’re not worried about eye contact and body language.’*

(GP 25)

There were also fewer children presenting with respiratory illnesses, which reduced the opportunity to use the CHICO intervention during the pandemic:

*‘We get a lot of virally coughs and colds and things in children but since lockdown and since COVID, there’s been hardly any and I suppose that’s because people aren’t going out and they’re not going to nurseries are they, and they’re not picking it up … we haven’t hardly any children in now.’*

(Nurse 20)

### Reflexive monitoring (appraisal of CHICO)

When appraising the CHICO intervention and making recommendations for future implementation, participants suggested expanding the template to encompass more information (for example, *‘physical examination findings like heart rate, respiratory rate’* [GP 13])*.* This could help overcome issues with having to record information in multiple places and switching screens:

*‘I think if you’re filling in a template, especially when we’re busy in the winter, it would be good if we could record all the information in that template and then not have to go back into the notes to record things that we think is important to record.’*

(GP 19)

Clinicians also recommended adapting the intervention to be more conducive to remote consultations. This could include informing carers about how to assess symptoms and having carer-reported criteria rather than having the clinician-assessed criteria. However, some clinicians worried about relying on carer-reported symptoms because these could be less accurate:

*‘I think the tool is little bit reliant on the clinical aspect as well, which you may not have, so it’s difficult to judge on chest signs and symptoms, and respiratory distress, and that sort of thing, wheeze, based on a conversation with a parent, and even temperature. They may not have a temperature probe so you may not be able to get particular aspects of it but then some bits you will be able to get. But if it can be tweaked to amend for things that may not happen on remote working then that may obviously help.’*

(GP 25)

Some participants valued the CHICO intervention and said they would use it in the future:

*‘I would have no problem with starting to use it again now because I feel you know, now we’re gonna start getting back to normal and coughs will just be coughs and colds, and it would be really useful to have that back again.’*

(Nurse 24)

## Discussion

### Summary

Clinicians initially welcomed the CHICO intervention in theory, but in practice it proved difficult to align the intervention flow with that of the clinician’s usual use of the computer during a consultation. GPs and nurses used the intervention at the start of the trial, but use waned over time. Most clinicians liked the algorithm template and found it straightforward to use, without adding any more time to consultations. However, having to close the patient’s record before the end of the consultation to complete the intervention process did not always align with their usual processes and was, therefore, problematic. The COVID-19 pandemic also impacted the use of the intervention as a result of changes to practice pathways, increased use of remote consultations, and reduced numbers of children presenting with RTIs.

While some clinicians reported that the intervention influenced their prescribing decisions and found it most useful in ‘borderline cases’ for hospitalisation and prescribing, others reported that they used it as a supportive aid during consultations rather than a tool to change prescribing behaviour. CHICO helped elicit carers’ concerns and reassure clinicians and carers of the appropriateness of some treatment decisions. Clinicians particularly liked the safety-netting carer advice leaflet, as it helped explain treatment decisions and home care with carers, and this was seen to be the most useful intervention component. To increase the use of the intervention, the findings suggest that it may need to be adapted for use in remote consultations and to fit better with clinicians’ consultation flow.

### Strengths and limitations

Strengths of this study include interviewing GPs and nurses who used the intervention who worked in a diverse range of practices. The use of normalisation process theory to inform data collection and analysis enabled a focus on issues with both the intervention design and the way it was implemented in practice.

A limitation is that most clinicians were interviewed towards the end of the trial, which may have been some time after they had last used the intervention. As a result, clinicians might not have recalled important aspects of intervention use. In addition, those clinicians who had never used the tool were not interviewed, and they could have provided useful insights into the barriers to using the tool, as could clinicians from a different selection of practices. RCT recruitment at the practice level limited the ability to interview CHICO-using carers, but clinicians provided valuable carers’ perspectives.

### Comparison with existing literature

Process evaluations of other interventions to reduce antibiotic prescribing have similarly found that clinicians value patient-facing materials and report that decision aids support rather than change their prescribing practice.^18^^,^^19^ Clinicians emphasise the need to educate parents and/or patients, and perceive their own prescribing practice to be clinically appropriate. However, the parent-oriented leaflet really acts as a tool to change clinicians’ behaviour, by providing them with a substitute for giving antibiotics.^8^ The safety-netting information in the leaflet, which was praised by the clinicians in this study, may also help because the clear advice means they feel safer not to prescribe.^20^ The content of the parent/carer leaflet was co-designed with parents/carers from a range of backgrounds, and the original version is available online on the University of Bristol website.^9^

The switch to online consultations during the COVID-19 pandemic might have made diagnosis and treatment decisions for children with RTIs more difficult.^21^ An in-person assessment of children plays a key role in clinicians’ diagnostic processes for children with RTIs.^22^ This study showed that many clinicians reported being unable to assess some of the symptomatic predictors of hospitalisation adequately. During the COVID-19 lockdowns, rates of RTIs reduced, but continued use of remote consultations for children could contribute to higher rates of antibiotic prescribing because of increased uncertainty, which is linked to high antibiotic prescribing.^5^^,^^20^

### Implications for research and practice

As found in the RCT, the CHICO intervention does not appear to change overall prescribing behaviour; however, it may still be effective in some clinical groups and it was found to be a useful tool for confirming clinical decision making. Therefore, clinicians may still find the intervention helpful to support decision making around antibiotic prescribing for children with RTIs and discussions with carers about their concerns and treatment decisions. The intervention may be most useful for patients who are considered to be borderline cases for hospitalisation risk.

The intervention may need to be adapted to align more with clinicians’ consultation flow and allow use during remote consultations to increase use. Patient electronic medical record providers could improve the effectiveness of the CHICO intervention if platforms could improve the timing of delivery of the intervention, such as decision aids appearing at the appropriate time in the consultation, and being aligned more with usual medical note taking.

Further research is needed to develop and evaluate effective electronic record-based antimicrobial stewardship interventions for children to help reduce unnecessary prescribing.
